# Evaluation of the Strength of Slab-Column Connections with FRPs Using Machine Learning Algorithms

**DOI:** 10.3390/polym14081517

**Published:** 2022-04-08

**Authors:** Nermin M. Salem, Ahmed Deifalla

**Affiliations:** 1 Electrical Engineering Department, Future University in Egypt (FUE), Cairo 11835, Egypt; nfawzy@fue.edu.eg; 2 Structural Engineering and Construction Management Department, Future University in Egypt (FUE), Cairo 11835, Egypt

**Keywords:** two-way shear, FRP, slab-column connection

## Abstract

Slab-column connections with FRPs fail suddenly without warning. Machine learning (ML) models can model the behavior with high precision and reliability. Nineteen ML algorithms were examined and compared. The comparisons showed that the ensembled boosted tree model showed the best, most precise prediction with the highest coefficient of determination (R^2^) (0.98), the lowest Root Mean Square Error (RMSE) (44.12 kN), and the lowest Mean Absolute Error (MAE) (35.95 kN). The ensembled boosted model had an average of 0.99, a coefficient of variation of 12%, and a lower 95% of 0.97, respectively, in terms of the measured strength. Thus, it was found to be more accurate and consistent compared to all implemented machine learning models and selected traditional models. In addition, the significance of various parameters with respect to the predicted strength was identified, where the effective depth was the most significant by a factor of 0.9, and the concrete compressive strength was the lowest by a factor of 0.3.

## 1. Introduction

Vital infrastructures suffer from the risk of demolition due to a lack of maintenance, severe environmental conditions due to steel corrosion, and the sudden nature of slab-column connection failures. Thus, fiber-reinforced polymers (FRPs) are replacing steel to avoid problems due to steel corrosion because of their excellent properties, which include, but are not limited to, being non-corrosive in nature, having a high strength-to-weight ratio, and performing well under fatigue [1,2,3,4,5]. It is worth noting that, back in the 90’s, FRPs were used for strengthening structures and continue to be valuable in this field [6,7]. In addition, most of the existing design models for slab-column connections lack a physical sense, which is due to their being empirical or semi-empirical [4]. On the other hand, machine learning (ML) models can model the behavior with a high level of precision and consistency [8,9,10].

Although the punching shear failure has a sophisticated, complex behavior, other innovative data-driven models are essential for improving prediction accuracy [11,12,13,14,15]. For the last few decades, ML has shown significant improvements in various fields [16,17,18,19], including structural engineering [20,21,22,23].

Some studies have tackled slab-column connections with FRP reinforcements. In [24], Jumaa and Yousif examined three ML prediction models, Nonlinear Regression analysis (NLR), an Artificial Neural Network model (ANN), and GEP to predict the punching shear failure of FRPs. The models were trained on a dataset composed of 269 records. The results showed that the ANN model outperformed the other two models regarding prediction accuracy. In [20], two models were presented, one based on an ANN and one based on an SVM; both models were trained using a dataset composed of 82 records. In [25], Metwally employed a Levenberg–Marquardt Artificial Neural Network (LM-ANN) for the prediction of the punching shear strength of concrete slabs with various types of FRPs. His model was trained on a small dataset composed of 59 records. His method showed promising results when compared to the experimental results.

All previous ML models, used for the punching shear of FRP-reinforced slabs that were examined, worked with a relatively small dataset. In our study, a comprehensive study was performed for five main ML algorithms, and all of our models were trained with a relatively large dataset, composed of 189 records. The dataset of the five ML algorithms, used for the prediction of the punching shear of FRP slabs, was divided into three subsets: training, validation, and testing. In addition, the effect of all input parameters on prediction was examined, and all of our models were compared with each other with respect to model efficiency and prediction accuracy. Several design models for slab-column connection design models are shown in Table 1. Models were selected to represent well-known simple design models, including, but not limited to, well-established design codes, guides, and recently developed models, with both empirical and semi-empirical designs. However, these models were developed using an old experimental database and thus lack the consistency needed for reliable strength prediction. In addition, these models vary in the variables considered and their patterns. Thus, there is a need for a machine learning model capable of accurately predicting strength and identifying the inter-relationships between variables. The selected models in Table 1 are being used for comparison with the proposed model as evidence of its accuracy and consistency.

**Table 1:** Selected slab-column strength models.

Design Model
JSCE [26] $V=\beta_{d}\beta_{\rho }\beta_{r}f_{Pcd}b_{0.5d}d$
$\beta_{d}={\left(\frac{1000}{d}\right)}^{1/4}\leq 1.5$, $\beta_{\rho }={\left(100\rho E/E_{s}\right)}^{1/3}\leq 1.5$, $\beta_{r}=1+\frac{1}{\left(1+\frac{{0.25b}_{0.5d}}{d}\right)}$, $f_{Pcd}=\;0.2\sqrt{f_{c}^{{}^{\prime }}}\;\leq 1.2$,
$b_{0.5d}=4\left(c+d\right)$
CSA [27] $V=b_{0.5d}d\left\{\begin{array}{c} 0.028\left(1+\frac{2}{\beta_{c}}\right){\left(E\rho f_{c}^{{}^{\prime }}\right)}^{1/3}\\ 0.147{\left(E\rho f_{c}^{{}^{\prime }}\right)}^{1/3}\left(0.19+\alpha_{s}\frac{d}{b_{0.5d}}\right)\\ 0.056{\left(E\rho f_{c}^{{}^{\prime }}\right)}^{1/3} \end{array}\right.$
$\beta_{c}=$ 1, $\alpha_{s}$= 4, 3, 3 for an inner, edge, corner connection, $b_{0.5d}=4\left(c+d\right)$
ACI [28] $V=0.8\sqrt{f_{c}^{{}^{\prime }}}kdb_{0.5d}$
$k=\sqrt{2\rho n+{\left(\rho n\right)}^{2}}-\rho n$, $n=\frac{E}{E_{c}}$, $E_{c}=4750\sqrt{f_{c}^{{}^{\prime }}}$, $b_{0.5d}=4\left(c+d\right)$
Hemzah [29] $V=\;\frac{1}{3}\sqrt{f_{c}^{{}^{\prime }}}k\left[{\left(\frac{90}{f_{c}^{{}^{\prime }}}\right)}^{0.33}{\left(5\rho \right)}^{0.39}{\left(\frac{E}{E_{s}}\right)}^{0.3}\right]b_{0.5d}d$
k=0.77and0.55 for circular and rectangular columns, respectively
Ju [30] $V=2.3\;{\left(100\rho \frac{E}{E_{s}}f_{c}^{{}^{\prime }}\right)}^{1/2}{{\left(\frac{d}{b_{0.5d}}\right)}^{1/2}b}_{0.5d}d$
$b_{0.5d}=4\left(c+d\right)$

## 2. Analysis of the Dataset

**Figure 1:**
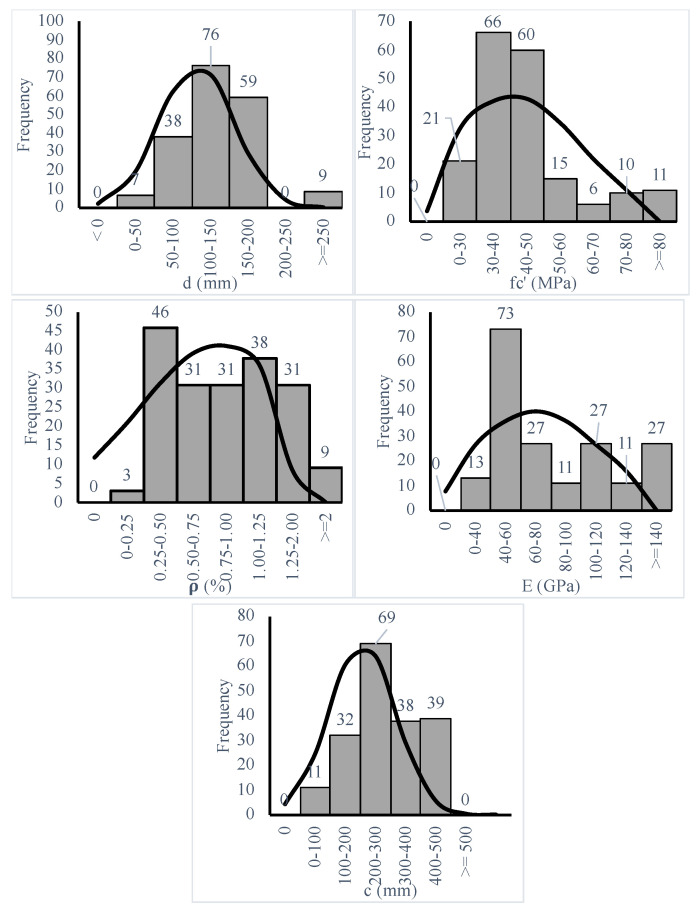
Frequency spectrum of different Inputs.

An extensive dataset of 189 records representing the test results of slab-column capacity with FRP slabs tested under punching shear was gathered from 37 research investigations, which will be referred to hereinafter as the dataset, as shown in Figure 1 and Table 2, where CFRP is carbon FRP, GFRP is glass FRP, N is the number of tested specimens, V is the punching shear failure load, E is Young’s modulus, d is the effective depth, fc’ is the concrete compressive strength, ρ is the flexure reinforcement ratio, b and c are the column dimensions, and A and B are the slab dimensions. In Figure 1, the dataset is close to normally distributed with respect to all variables. In Figure 1 and Table 2, the dataset covered a range with respect to all significant variables, including, but not limited to, the following:

1. Slab dimensions vary from 300 to 4000 mm.
2. Effective depth varies from 45 to 284 mm, while there are very few specimens above 200 mm, which is not common for a flat slab; however, this could be because of the lab testing facility.
3. Concrete compressive strength varies from 22 (conventional normal concrete) to 179 MPa (ultra-high-performance concrete), while there are very few specimens above 50 MPa. Thus, there is a need for more testing of high strength concrete and ultra high strength concrete.
4. The flexure reinforcement ratio varies from 0.18% to 3.26%, which is a wide range of ratios.
5. Young’s Modulus varies from 28 to 230 GPa, and the majority of values are between 40 and 60 MPa. However, the FRP industry is evolving with new products with much higher Young’s modulus values. Thus, more testing of FRP reinforcements with a Young’s modulus up to the maximum values offered by the market is needed.
6. The shear-span-to-depth ratio varies between 1.8 and 11.
7. The loading area dimensions vary from 25 to 635 mm.

FRP reinforcements have different diameters and configurations, where the diameter varies between 6 to 24 mm and the configurations are bars or grids. However, the flexure reinforcement ratio and Young’s modulus were implemented to consider the influence of FRP material type, diameter, and configuration.

**Table 2:** Experimental database for FRP-reinforced concrete slab-column connections.

Reference	n	A	B	b	c	d	*f* c’	ρ	E	V	Type
		(mm)	(mm)	(mm)	(mm)	(mm)	(MPa)	(%)	(GPa)	(kN)	
**Ahmed et al. (1993) [31] **	4	690	690	75–100	75	61	36–45	0.95	113	78–99	CFRP
**Banthania et al. (1995) [32]**	3	600	600	100	100	55	41–53	0.31	100	61–72	CFRP
**Bank and Xi (1995) [33]**	6	1800	1500	250	250	76	30	1.49–2.05	143–156	179–201	CFRP
**Louka (1999) [34]**	12	3000	1800	575	225	175	43–55	1	39–160	500–1183	GFRP, and CFRP
**Matthys and Tarewe (2000) [35]**	13	1000	1000	80–230	80–230	95–126	32–118	0.19–1.22	37–149	142–347	CFRP and GFRP
**Rahman et al. (2000) [36]**	5	2000	2500	250	150	162	42	0.28	85	534–698	GFRP
**Hassan et al. (2000) [37]**	3	1800	3000	575	225	165	59	0.57	147	1000–1328	CFRP
**Khanna et al. (2000) [38]**	1	2000	4000	500	250	138	35	2.4	42	756	GFRP
**El–Ghandour et al. (2003) [39]**	5	2000	2000	200	200	142	29–47	0.18–0.47	45–110	170–317	GFRP and CFRP
**Ospina et al. (2003) [40]**	3	2150	2150	250	250	120	29.5–37.5	0.73–1.46	28–34	206–260	GFRP
**Zaghloul and Razapur (2003) [41]**	1	1760	1760	250	250	75	45	1	100	234	CFRP and GFRP
**Hussien et al. (2004) [42]**	4	1830	1830	250	250	100	26–40	1.05–1.67	42	210–249	GFRP
**Jacobson et al. (2005) [43]**	5	2000–2300	2000	635	250	175	27.6	0.95–0.98	33	537–897	GFRP
**El–Gamal et al. (2005) [44]**	5	3000	2500	600	250	159	44–49.6	0.35–1.99	38–122	674–799	GFRP and CFRP
**Zhang et al. (2005) [45]**	2	1830	1830	250	250	100	35–71	1.05–1.18	42	218–275	GFRP
**Zhang (2006) [46]**	7	1900	1900	250	250	100	25–98	0.36–0.75	120	251–446	CFRP
**Tom (2007) [47]**	6	1900	1900	250	250	110	70	1–1.5	41	282–487	GFRP
**Zaghloul (2007) [48]**	7	1760	1000	250	250	120	25	0.94–1.48	100	97–211	CFRP
**El–Gamal et al. (2007) [49]**	2	3000	2500	600	250	156	44.1	1.2	44.5	707–735	GFRP
**Ramzy et al. (2007) [50]**	4	2000	2000	200	200	82–112	33–40	0.81–1.54	46	165–230	GFRP
**Zaghloul et al. (2008) [51]**	4	1760	1760	200	200	82–112	33–40	0.81–2.14	46	165–230	GFRP
**Lee et al. (2009) [52]**	4	2300	2300	225	225	110	36.3	1.17–3	48.2	222–330	GFRP
**Zhu (2010) [53]**	7	1500	1500	150	150	135	22–42	0.29–0.55	100	145–275	BFRP
**Min (2010) [54]**	7	300	300	25	25	45	47.8–179	0.78	76–230	39–98	GFRP and CFRP
**Bouguerra et al. (2011) [55]**	7	3000	2500	600	250	110–155	35–65	0.70–1.20	43	362–732	GFRP
**Zhu et al. (2012) [56]**	5	1500	1500	150	150	130	22–45	0.29–0.55	45.6	167–252	GFRP
**Nguyen–Minh et al. (2013) [57]**	3	2200	2200	200	200	130	48.8	0.48–0.92	48	180	GFRP
**Hassan et al. (2013) [58]**	19	2500	2500	300	300	131–284	32–75	0.30–1.61	48–57	329–1248	GFRP
**El-Gendy et al. (2015) [59]**	6	2800	1500	300	300	160	41	0.85–1.70	60.5	159–277	GFRP
**Tharmarajah et al. (2015) [60]**	4	1425	500	500	25	117–119	65–69	0.6	54–67.4	295–365	GFRP
**Mostafa et al. (2016) [61]**	3	2600	1450	300	300	160	80–85	0.87–1.70	60.5–69.3	251–288	GFRP
**ELGABBAS (2016) [62]**	6	3000	2000	600	250	160	42–48	0.40–1.20	69.3	436–716	BFRP
**Gouda and El–Salakawy (2016) [63]**	4	2600	2600	300	300	160	38–70	0.65–1.30	65–69	363–719	GFRP
**Oskouei et al. (2017) [64]**	1	800	800	250	250	176	59	0.7	68	719	GFRP
**Hussein and El–Salakawy (2018) [65]**	3	2800	2800	300	300	160	80–87	0.98–1.93	65	461–604	GFRP
**Hemzah et al. (2019) [29]**	8	600	600	100	100	80	46–60	0.3–0.90	144	57–129	CFRP
**Huang et al. (2020) [66]**	1	1600	1600	200	200	125	24.97	0.89	123	262	CFRP
**Mean**		1961	1736	301	212	131	46	0.94	80	416	
**Minimum**		300	300	25	25	45	22	0.18	28	39	
**Maximum**		3000	4000	635	300	284	179	3.76	230	1600	

## 3. Machine Learning Methods

Before the implementation phase of the five ML models, dividing the dataset into two subsets, a training set with 80% of the dataset, with a handout validation of 15%, and a testing set with the remaining 20%, is recommended in order to determine the best-fit model. The testing dataset was not used in the training phase. The models were trained with five input variables: the column dimension C, the effective depth d, the concrete compressive strength f’c, the flexure reinforcement ratio ρ, and young’s modulus E. The output of our models is the prediction of the slab-column strength of FRP V. The evaluation of the five models was performed on the testing set.

### 3.1. Linear Regression Model

This model is defined as a linear fit regression that interprets the relationship between the output and the influencing inputs. The key concept is to indicate the coefficient parameters’ linearity. Different types of linear regression were examined, including normal, interaction, robust, and step-wise.

### 3.2. Regression Decision Tree

Decision trees subdivide data in a practical tree illustration using simple rules [67]. These rules are set through the decision tree, and the response prediction is performed in an iterative segmentation manner. The tree is composed of roots, leaves, and branches. The training dataset is arranged at the bottom of the tree. The training starts from the top-most roots of the tree. Afterwards, a conditional test is performed in order to draw a path along the tree branches for every node. Various judging conditions are applied to assess the testing at each node, such as the Mean Square Error (MSE). The output of the tree is the prediction that exists in the leaves of the tree, at the end of each path. Various types of regression trees are examined, including complex trees, medium trees, and simple trees. They all follow the same concept of prediction; however, they differ in the fixation of the minimum size of the leaf.

### 3.3. Ensemble Trees

The ensemble method was introduced in [68] as a group of separate, inadequate models that provide a powerful mathematical prediction. These types of trees can combine similar or non-similar prediction algorithms. There are two types of ensemble trees: bagged and boosted trees. The bagged trees create many models by implementing various bootstraps in a single tree and then merging them into single decision tree by computing the average between them. Boosted trees work as a two-step technique. In the first step, a subset of data is used to obtain a sequence of average working models; in the second step, the performance is boosted by joining the models with each other using a fixed cost function. The boosted algorithm relies on an iterative approach, which means that the parameters in the next step are updated using the residual computed from the previous step in order to optimize the objective function, which is defined as

(1) $$J\left(\theta \right)=L\left(\theta \right)+\;\Omega \left(\theta \right)$$

(2) $$L\left(\theta \right)=\;\sum_{i=1}^{n}L(y_{i},{\;\hat{y}}_{i})$$

(3) $$\Omega \left(\theta \right)=\;\sum_{k=1}^{m}\Omega \left(f_{k}\right)$$

(4) $$\Omega \left(f_{k}\right)=\;\Upsilon T+\frac{\lambda }{2}\sum_{j=1}^{T}w_{j}^{2}$$

where θ is the trained parameters among the given data, J(θ) is the objective function, $L\left(\theta \right)$ is the training loss function, which is computed through the comparison between the predicted output ${\;\hat{y}}_{i}\;$and the real output $y_{i}$ to evaluate the accurate prediction of the model, $\Omega \left(\theta \right)$ is the regularization term, which is added to prevent model over-fitting by controlling the complexity of the algorithm, n and m are the number of predictions and trees, respectively, $f_{k}$ is the individual tree prediction function to evaluate the output in the functional space F of all regression trees, Υ and λ are the regularization parameters terms, also used in controlling the complexity of the boosted algorithm, T is the number of tree leaf nodes, and $w_{j}$ is the weight of the ith leaf node.

The prediction result ${\;\hat{y}}_{i}$ of the boosted algorithm is computed from the prediction of each individual tree, defined as

(5) $${\hat{y}}_{i}=\;\sum_{k=1}^{m}f_{k}\left(x_{i}\right),\;\;f_{k}\in F\;$$

where $x_{i}$ is the ith input variable.

The boosted algorithm constructs a tree and splits a leaf node into two sub-tresses/ branches, left and right. Afterwards, the gain is computed at each leaf node for the determination of the best node. The optimal branch gain is selected once the gain after the splitting reaches its maximum value.

(6) $$Gain=\frac{1}{2}\left[\frac{G_{R}^{2}}{H_{R}+\lambda }+\frac{G_{L}^{2}}{H_{L}+\lambda }-\frac{{{(G}_{R}^{2}+G_{L}^{2})}^{2}}{{H_{R}+H}_{L}+\lambda }\right]-\;\Upsilon$$

where $G_{R}$ and $G_{L}$ are the gain of the new right and left branches, respectively, and $H_{R}$ and $H_{L}$ are the original right and left branches, respectively.

The boosted algorithm showed the best fit model for the prediction for FRP capacity with respect to the available dataset.

### 3.4. Support Vector Machine (SVM)

The concept of a SVM was first introduced in [69]. It is a method of implementing kernel functions for transforming data into a high dimensional feature space through a linear model. This model is utilized to remove any sophisticated nonlinear relationship. The main concept underlying the SVM is the linear regression function calculations, where the input data is mapped using a nonlinear function.

The training in the regression process can be defined as {($x_{1},\;y_{1}$), ($x_{2},\;y_{2}$), ...., ($x_{n},\;y_{n}$)}, where $x_{i}$ and $y_{i}$ are the input vector and predicted output value of the SVM models, respectively, and n is the size of the sample. The aim is to find the function $f(x_{i})$ that has the maximum derivation ε of the real output $y_{i}$ for all samples in the training set. f(x) is assumed to have a linear regression function that can be defined as

(7) $$f\left(x\right)=\langle w,x\rangle +b$$

where the vector w is required to be minimized to allow the function f(x) to be as flat as possible. This minimization is achieved by computing the norm $\frac{{∥w∥}^{2}}{2}$ subject to $\left|y_{i}-\langle w,\;x_{i}\rangle -b\right|\leq \varepsilon$.

Some points in the training data may not satisfy the constraint condition. In this case, a slack variable $\varphi_{i}$ will be introduced for such a sample to be able to deal with the constraint condition. This slack variable $\varphi_{i}$ will measure the derivation of the training points outside the εsupported points. Therefore, the SVM function will be computed to minimize as follows:

(8) $$\frac{{∥w∥}^{2}}{2}+C\sum_{i=1}^{n}\varepsilon_{i}:\left\{\begin{array}{c} \left\lceil \left|y_{i}-\langle w,\;x_{i}\rangle -b\right|\leq \varepsilon_{i}+\varphi_{i}\right\rceil \\ \varphi_{i}\;\geq 0 \end{array}\right.$$

where C > 0 is the regularization parameter and is responsible for the determination of the trade-off between the function f(x) and the calculated error.

The final estimation function for to minimize:SVM is recalculated to be

(9) $$f\left(x\right)=\;\sum_{i=1}^{n}\sigma_{i}K\left(X,\;x_{i}\right)+b$$

where $\sigma_{i}$ is the Lagrange multiplier, $K\left(X,\;x_{i}\right)$ is the kernel function, and b is the bias term.

Many SVM approaches have been examined, including linear, quadratic, cubic, fine, medium, and coarse SVMs. They all follow the same concept but have different kernel functions.

### 3.5. Gaussian Process Regression

Gaussian Regression (GPR) was introduced in [64]. It is a complex model that is capable of solving sophisticated ML problems. The power of such a model is its flexible, non-parametric models. GPR models are able to analysis the smoothness and noise parameters from the training data. The models find a stochastic process in which random variables are assumed to follow a Gaussian distribution. GPR models are non-parametric kernel-based probabilistic supervised learning models used for generalizing a complex and nonlinear function mapping hidden in the datasets used in training. The power of GPR methods is based on the use of kernel functions that improve efficiency when handling nonlinear data. GPR models provide a reliable response to the input data. These models assume that the output is computed as

(10) $$y=f\left(x\right)+\varepsilon$$

where ε is the noise representation of sample $x_{i}$.

For a given training set, the main goal is to predict the output value y${}^{*}$ of a new input pattern. In order to be able to establish this goal, it is essential to establish three co-variance matrices as follows:

(11) $$K=\left[\begin{array}{ccc} k(x_{1},x_{1}) & k\left(x_{1},x_{2}\right)\cdots & k(x_{1},x_{n})\\ k(x_{2},x_{1}) & k\left(x_{2},x_{2}\right)\cdots & k(x_{2},x_{n})\\ \cdots & \cdots & \cdots \\ \cdots & \;\;\;\;\cdots \;\;\;\;\; & \cdots \\ k(x_{n},x_{1}) & k\left(x_{n},x_{2}\right)\cdots & k(x_{n},x_{n}) \end{array}\right]$$

(12) $$K*=\left[\begin{array}{ccc} k(x_{*},x_{1}) & k(x_{*},x_{2})\cdots & k(x_{*},x_{n}) \end{array}\right]$$

(13) $$K**\;=\;k(x_{*},x_{*})$$

where $k(x_{1},x_{1})$ is the co-variance function, which maps the relation between one output and the next.

Many GPR methods have been examined, including the squared Gaussian process, the Marten 5/2 GPR, the exponential GPR, and the rational quadratic GPR.

## 4. Results and Discussion

To develop the used ML-based models, a grid search method with a 15-fold cross-validation approach was used in the training phase to determine the optimal hyper-parameters. In order to evaluate the effectiveness of our models, the following statistical measures were reported:

- Correlation Coefficient (R^2^), defined in Equation (14);
- Root Mean Square Error (RMSE) measured in kN, defined in Equation (15);
- Mean Absolute Error (MAE) in kN, defined in Equation (16);
- The model’s training time, measured in seconds.

Models were trained using an Intel core i5, 8GB RAM, using the MATLAB 2021a Machine Learning toolbox. In addition, the R^2^, RMSE, and MAE values were calculated for each model as shown in Table 3, which will be discussed in this section.

(14) $$R^{2}=1-\frac{\sum_{i=1}^{m}{\left(Y_{p}-Y_{o}\right)}^{2}}{\sum_{i=1}^{m}Y_{o}-\frac{1}{m}\sum_{i=1}^{m}Y_{o}}$$

(15) $$\mathrm{RMSE}=\;\sqrt{\frac{1}{m}\sum_{i=1}^{m}{\left(Y_{p}-Y_{o}\right)}^{2}}$$

(16) $$\mathrm{MAE}=\;\frac{1}{m}\sum_{i=1}^{m}\left|Y_{p}-Y_{o}\right|$$

### 4.1. Linear Regression

The stepwise model had the highest R^2^ and the lowest RMSE and MAE, with values of 0.95, 69.438, and 55.976, respectively, in training. However, the model produced the worst values in testing. The stepwise model reported the lowest R^2^ and the highest RMSE and MAE, with values 0.64, 266.688, and 153.129, respectively, among other linear regression models. This could be because the testing set was not used in training and thus provided value ranges for the input patterns that were different from the values used in training. This means that this model was not able to produce accurate predictions.

### 4.2. Tree

Three different approaches were employed for solving the slab-column strength problem. The fine tree method performed best among the other tree methods in training and testing. It reported the highest R^2^ and the lowest RMSE and MAE, with values of 0.94, 75.741, and 52.98, respectively, in training and 0.84, 85.3446, and 60.8346, respectively, in testing. It also consumed the most training time among the different tree approaches. The fine tree method could be used for prediction; however, it is not the optimal solution for our problem. The worst tree method in training was the coarse tree, while the medium tree reported the worst values in testing. This means that these tree methods may not be reliable enough for solving such a problem.

### 4.3. Support Vector Machine

Several SVM methods were examined for the prediction of the punching shear. The medium Gaussian SVM performed the best among the other tree methods in training and testing. It reported the highest R^2^ and the lowest RMSE and MAE, with values of 0.96, 57.815, and 46.092, respectively, in training. I was not able to produce the same good results for testing, reporting 0.69, 236.587, and 109.372, respectively. This means that this method may not be able to produce accurate predictions for our problem.

### 4.4. Ensembled Trees

The boosted model reported the most optimal values for R^2^, RMSE, and MAE in both training and testing phases. It reported vales of 0.98, 44.12, and 39.95, respectively, in training and 0.97, 71.963, and 43.452, respectively, in testing. This means that this is the most optimal and powerful method that can capture all of the changes in the input parameter patterns and provide accurate predictions of the punching shear. The bagged methodology also gave good results in both training and testing, but it was not the most optimal and consumed more training time.

### 4.5. Gaussian Process Regression

GPR methods performed well in both training and testing phases. They provided a solution that was close to the optimum boosted ensembled tree solution but with double training time. The exponential GPR reported the highest R^2^ and the lowest RMSE and MAE, with values of 0.96, 60.245, and 43.738, respectively, in training and 0.93, 67.839, and 43.738, respectively, in testing, compared with the other GPR methods.

**Figure 2:**
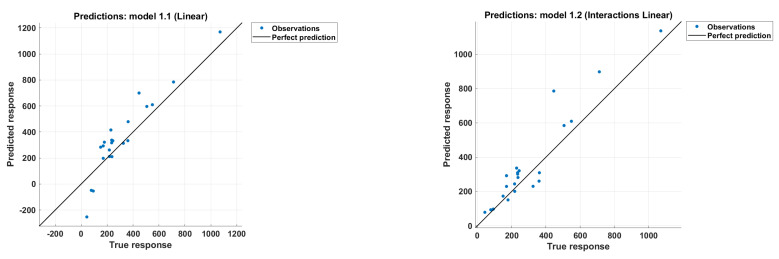

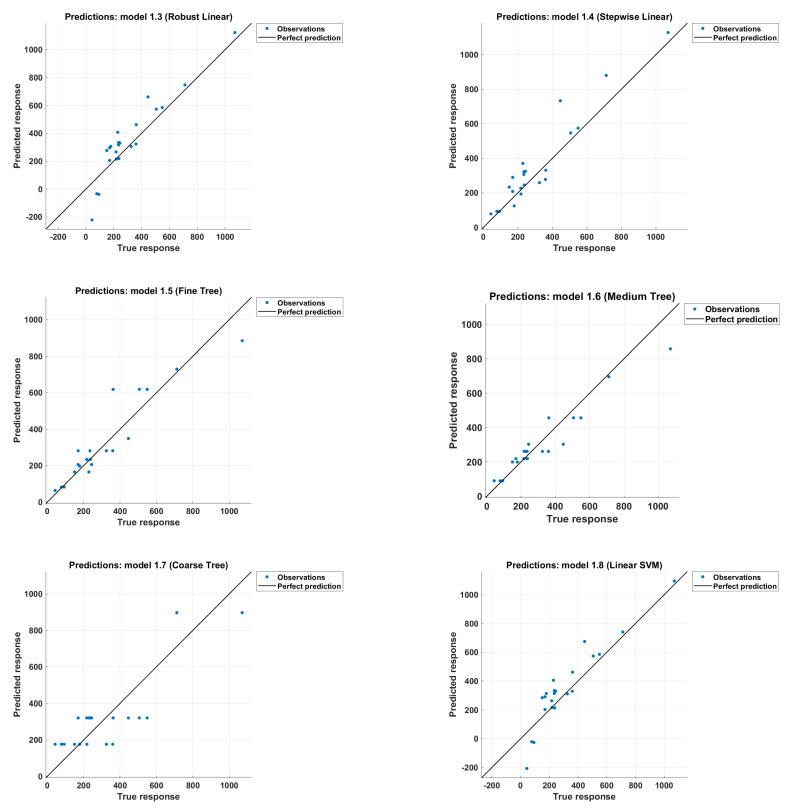

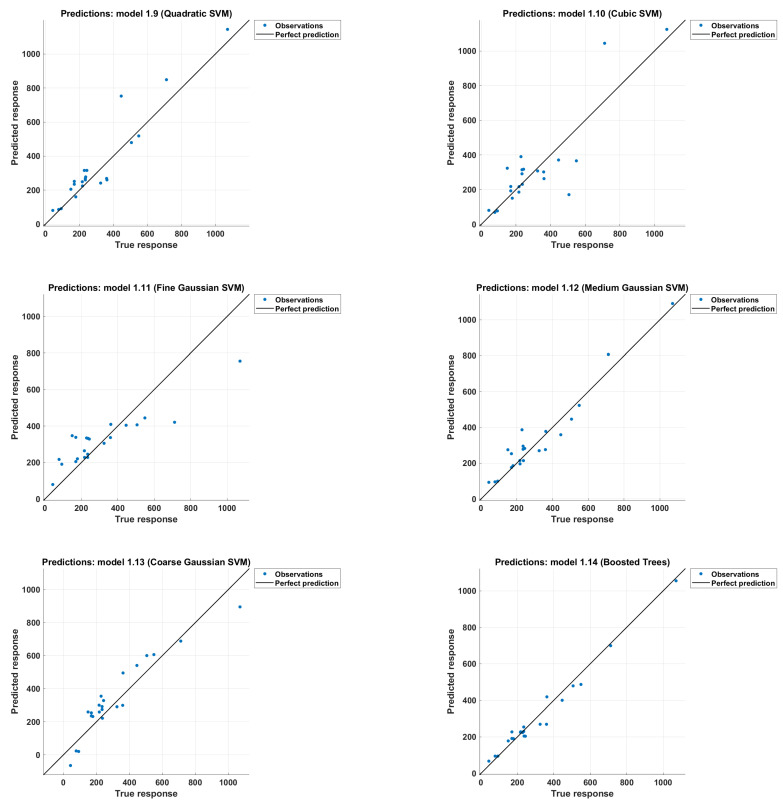

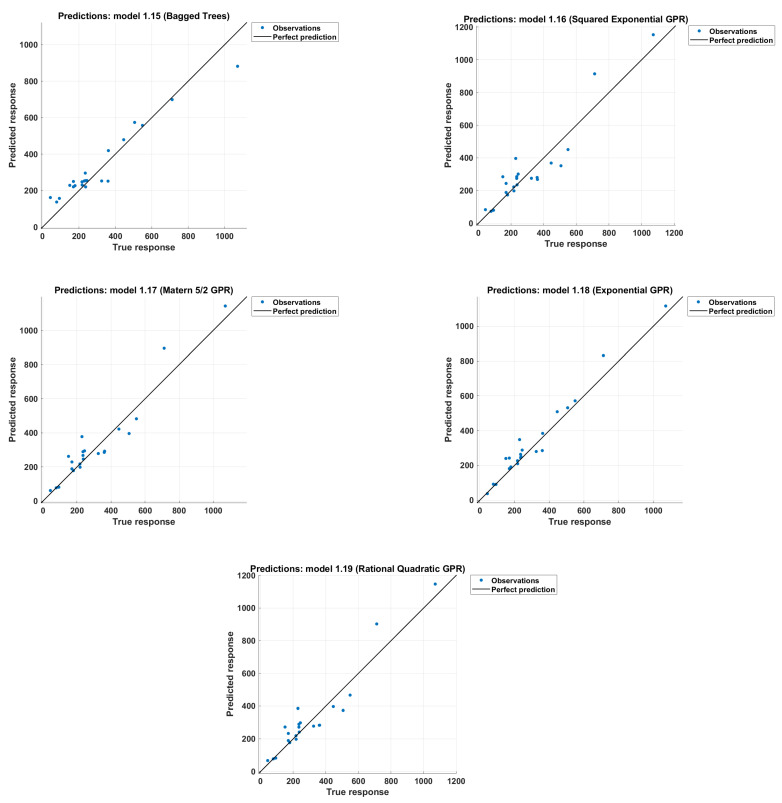
SF calculated using various models versus the record number.

**Figure 3:**
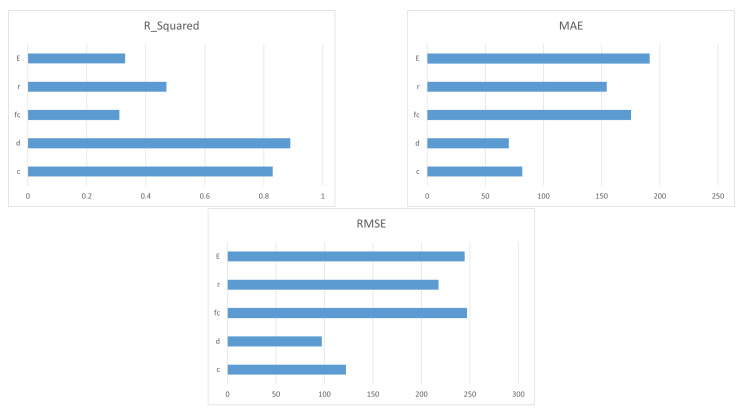
Importance of input variables in the boosted model reported in R^2^, MAE, and RMSE.

Figure 2 shows the predicted vs. actual values of the punching shear. This figure shows how well each model produces predictions for different response values. A perfect regression model has a predicted response equal to the actual response, where all of the points lie in the diagonal model, but the vertical distance from the line to any point is the error in the prediction of that point. Based on Figure 2, the best optimal solution of our problem was Model **1.14**—the boosted ensembled tree model. Almost all points either have the smallest distance with respect to the vertical line or lie on the vertical line itself, i.e., a zero error between the predicted and real output values, which is the best case. Figure 2 is generic, but further specifications regarding model performance are found in Table 3, where the R^2^, RMSE, and MAE values are reported for all models. We also investigated the effect of each of the five inputs on the most optimal model, the boosted ensembled tree. The analysis showed that the effective depth d had the most important effect on the prediction of the slab-column strength, i.e., it has highest R^2^ and the lowest RMSE and MAE, followed by column dimension C, while Youngs’ modulus E had the least important effect, i.e., the lowest R^2^ and the highest RMSE and MAE, as shown in Figure 3. The values are reported with respect to R^2^, MAE, and RMSE.

**Figure 4:**
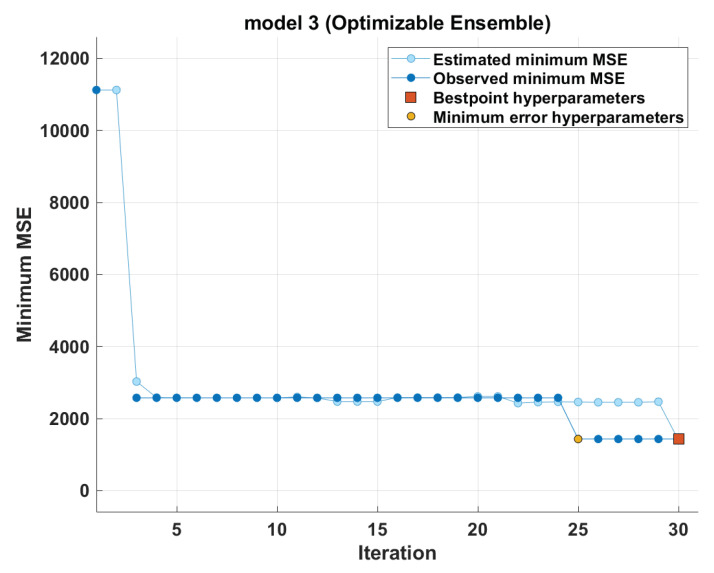
Visualization of the most optimal method—the boosted tree.

For the optimal method, the boosted tree in our case, Figure 4 shows how the error decreases as different combinations of hyper-parameters are evaluated. It also shows the model behaviour with the hyper-parameters that are optimized best. The best model converges in the 30th training iteration. It achieved the best performance in its 25th iteration.

## 5. Precision and Reliability of ML and Existing Models

In this section, the reliability and precision of the proposed mode will be compared to the most recent models, including, but not limited to, the Ju model developed in 2021 and the Hemzah developed in 2018. The precision and reliability of the capacity calculated using a model will be examined using the ratio between the experimentally observed capacity and that determined using that model (SF). Applying statistical measures on the SF calculated using the ML models and the existing ones could express the precision, reliability, and safety of the model. The closer the average of the SF is to unity, the more precise the used model is. The lower the coefficient of variation of the SF is, the more reliable and precise the model is. If the lower 95% confidence limit is close to unity and larger than 0.85, the model has acceptable safety. In addition, the SF can be plotted versus all effective parameters; thus, the variation in model safety can be examined. Moreover, the ideal pattern is plotted using a solid line, and a linear trendline for the SF is plotted using a dotted line. The inclination of the trendline is an indication of variation and scattering, while the sign of that inclination is indicative of an increase or decrease with the investigated parameter.

### 5.1. Overall Safety

**Figure 5:**
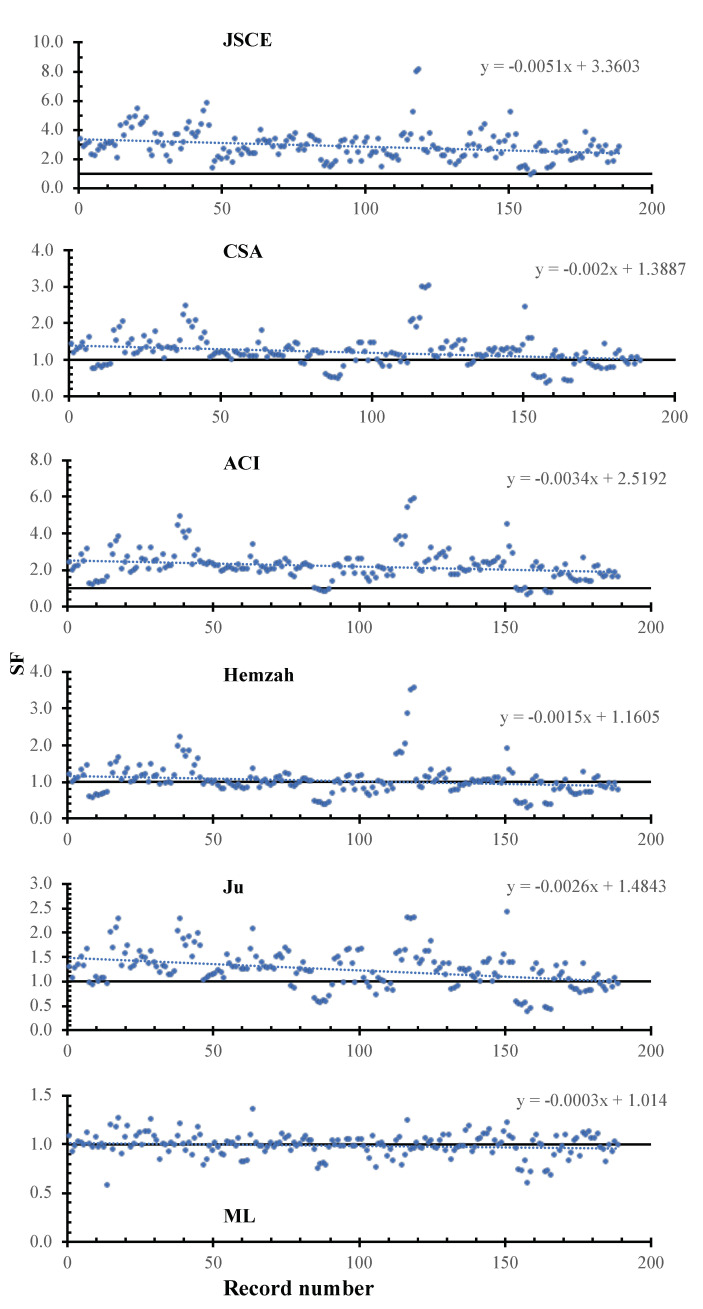
SF calculated using various models versus the record number.

Table 4 shows the statistical measures calculated for the existing models as well as the proposed model. It is clear that, overall, the ML model captured the behavior with a significantly lower RMSE, MAE, and coefficient of variation compared to all existing models, with values of 64.23, 37.97, and 12%, respectively. In addition, the proposed ML model is more precise, is more reliable, and is reasonably safe compared to the existing design codes in terms of the mean, R^2^, and the lower 95%, which is close to unity, with values of 0.96, 0.99, and 0.97, respectively. Excluding the ML model, the CSA, Hemzah, and Ju models are more reliable and accurate compared to the ACI and JSCE. Figure 5 shows the SF calculated using the JSCE, CSA, ACI, Hemzah, Ju, and ML models. In the figure, the inclinations of the trend line for the JSCE, CSA, ACI, Hemzah, and Ju models were $-51\times 10^{-4}$, $-20\times 10^{-4}$, $-34\times 10^{-4}$, $-15\times 10^{-4}$, $-26\times 10^{-4}$, and $-3\times 10^{-4}$, respectively. It is clear that the ML model has less scattering compared to the selected existing models.

### 5.2. Safety versus Slab Size

**Figure 6:**
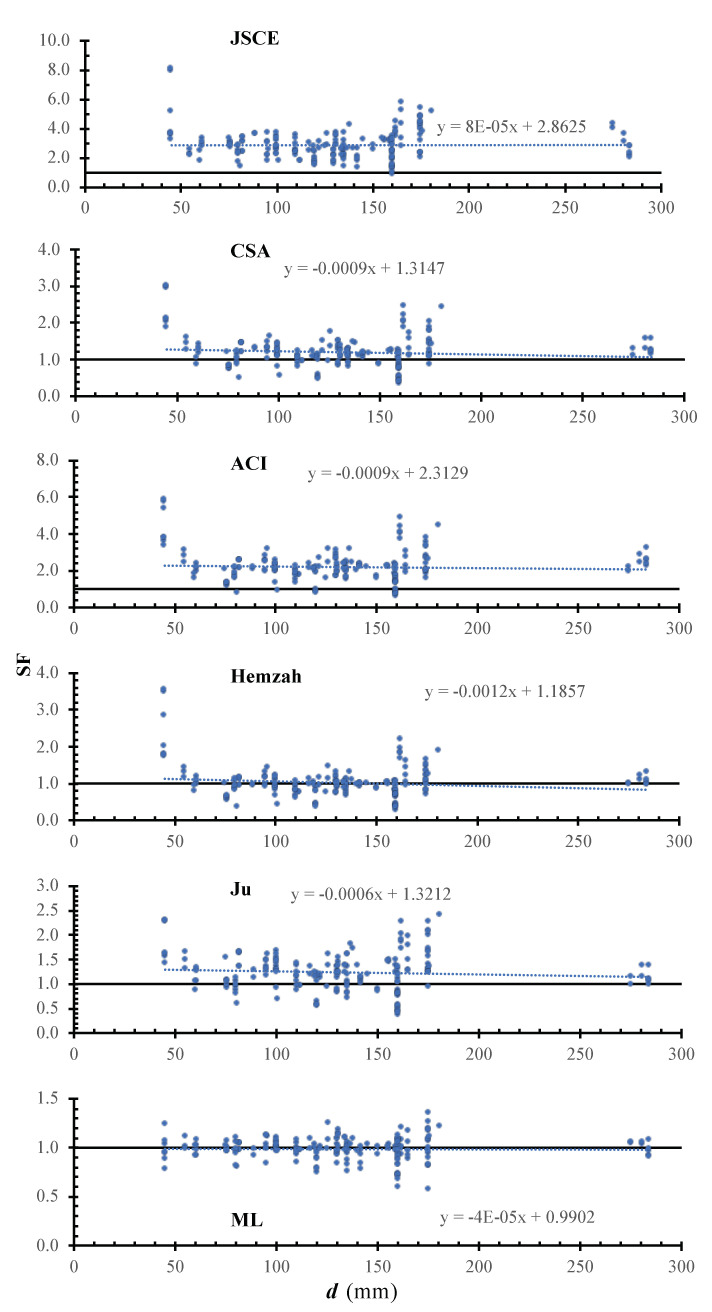
SF calculated using various models versus d.

Figure 6 shows the SF calculated using the JSCE, CSA, ACI, Hemzah, Ju, and ML models versus the effective depth. In the figure, the inclinations of the trend line for the JSCE, CSA, ACI, Hemzah, and Ju models was $0.8\times 10^{-4}$, $-9\times 10^{-4}$, $-9\times 10^{-4}$, $-12\times 10^{-4}$, $-6\times 10^{-4}$, and $-0.4\times 10^{-4}$. For all models, the safety decreases with the depth increase, except in the case of the JSCE and CSA models. The ML has less scattering compared to the other models. The JSCE and Ju models have a small inclination, which might be due to the size effect factor.

### 5.3. Safety versus Concrete Strength

**Figure 7:**
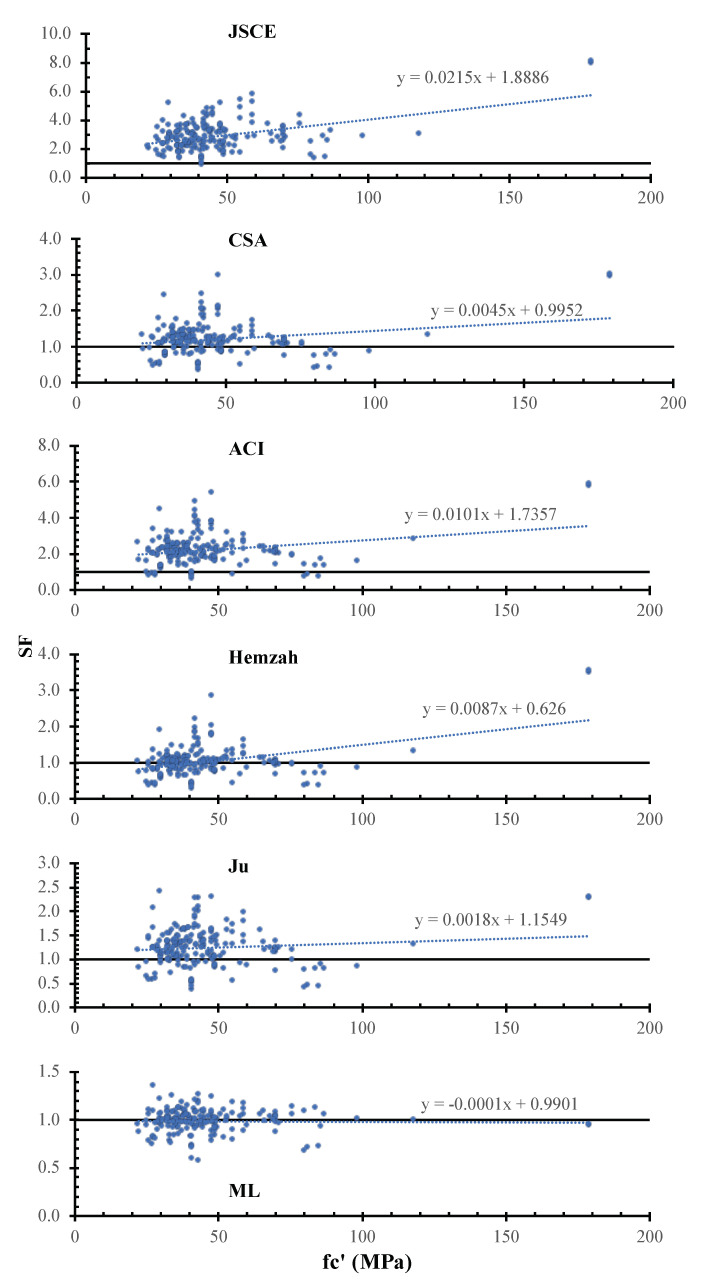
SF calculated using various models versus d.

Figure 7 shows the SF calculated using the JSCE, CSA, ACI, Hemzah, Ju, and ML models versus the concrete strength. In the figure, the inclinations of the trend line for the JSCE, CSA, ACI, Hemzah, and Ju models were $215\times 10^{-4}$, $45\times 10^{-4}$, $100\times 10^{-4}$, $-87\times 10^{-4}$, $18\times 10^{-4}$, and $-1\times 10^{-4}$. For all models, the safety increases with the concrete compressive strength increase, except in the case of ML. The ML model has less scattering compared to the other models.

### 5.4. Safety versus the FRP Young’s Modulus

**Figure 8:**
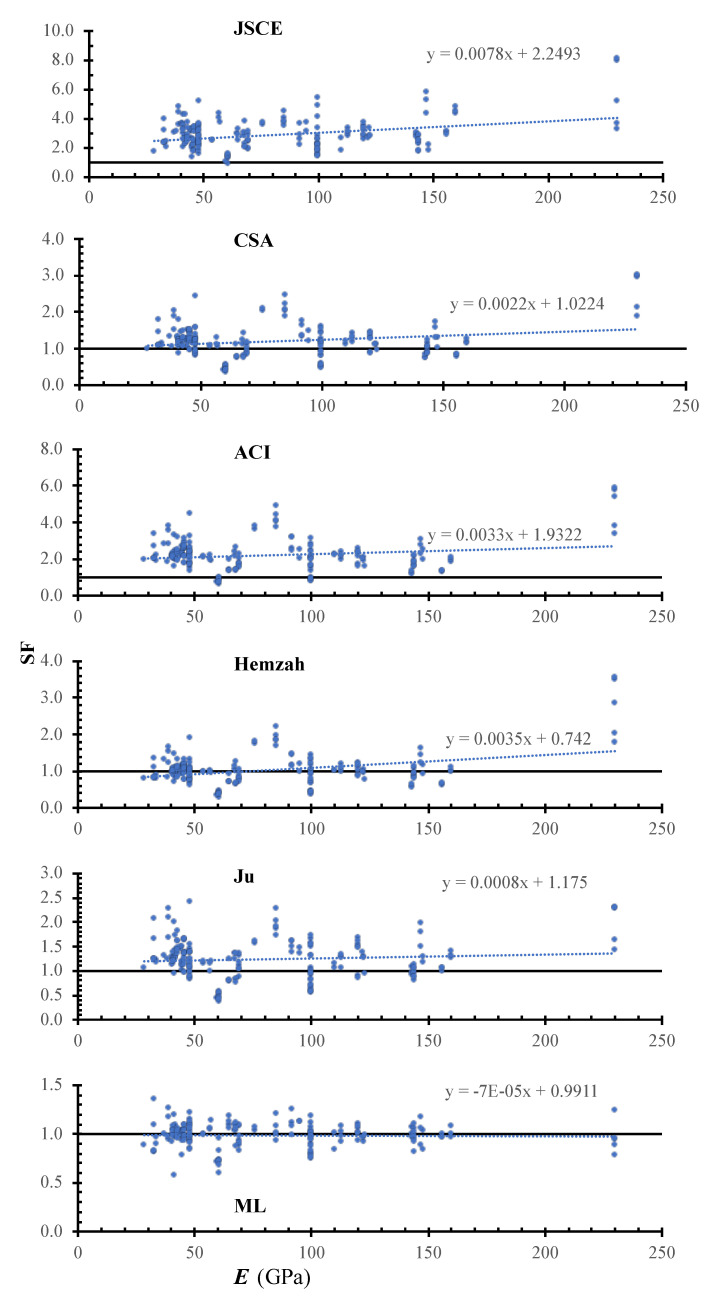
SF calculated using various models versus Γ.

Figure 8 shows the SF calculated using the JSCE, CSA, ACI, Hemzah, Ju, and ML models versus the concrete density. In the figure, the inclinations of the trend line for the JSCE, CSA, ACI, Hemzah, and Ju models were $78\times 10^{-4}$, $22\times 10^{-4}$, $33\times 10^{-4}$, $35\times 10^{-4}$, $8\times 10^{-4}$, and $-0.7\times 10^{-4}$. For all models, the safety increases with the increase in Young’s modulus, except in the case of the ML. The ML has less scattering compared to the other models. The Ju model has a small inclination, which might be due to the use of a square root relation, while other models use a cubic root relation.

### 5.5. Safety versus Column-Dimension-to-Depth Ratio

**Figure 9:**
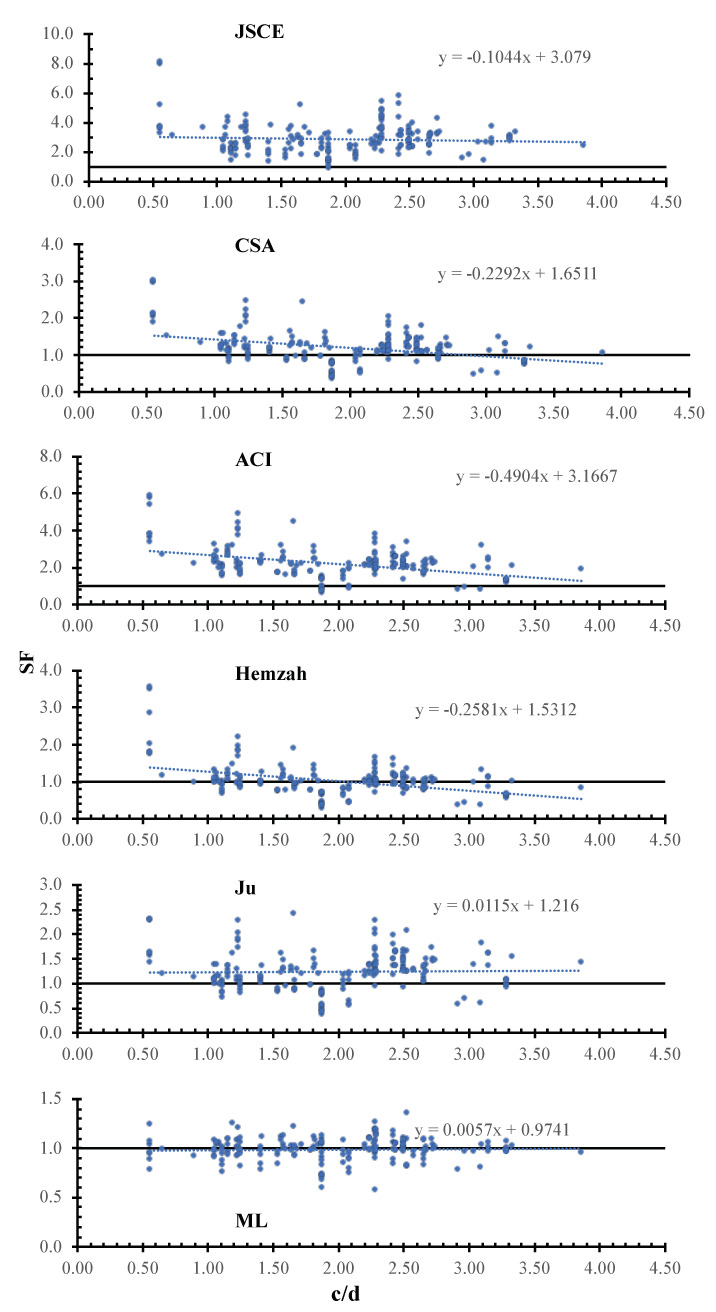
SF calculated using various models versus $\frac{c}{d}$.

Figure 9 shows the SF calculated using the JSCE, CSA, ACI, Hemzah, Ju, and ML models versus the column dimensions. In the figure, the inclinations of the trend line for the JSCE, CSA, ACI, Hemzah, and Ju models were $-1044\times 10^{-4}$, $-2292\times 10^{-4}$, $-4904\times 10^{-4}$, $-2581\times 10^{-4}$, $115\times 10^{-4}$, and $57\times 10^{-4}$. The ML has less scattering compared to the other models. The Ju model has a small inclination with respect to column dimensions, which might be due to the factor ${\left(\frac{d}{b_{0.5d}}\right)}^{0.5}$.

### 5.6. Safety versus Flexure Reinforcements

**Figure 10:**
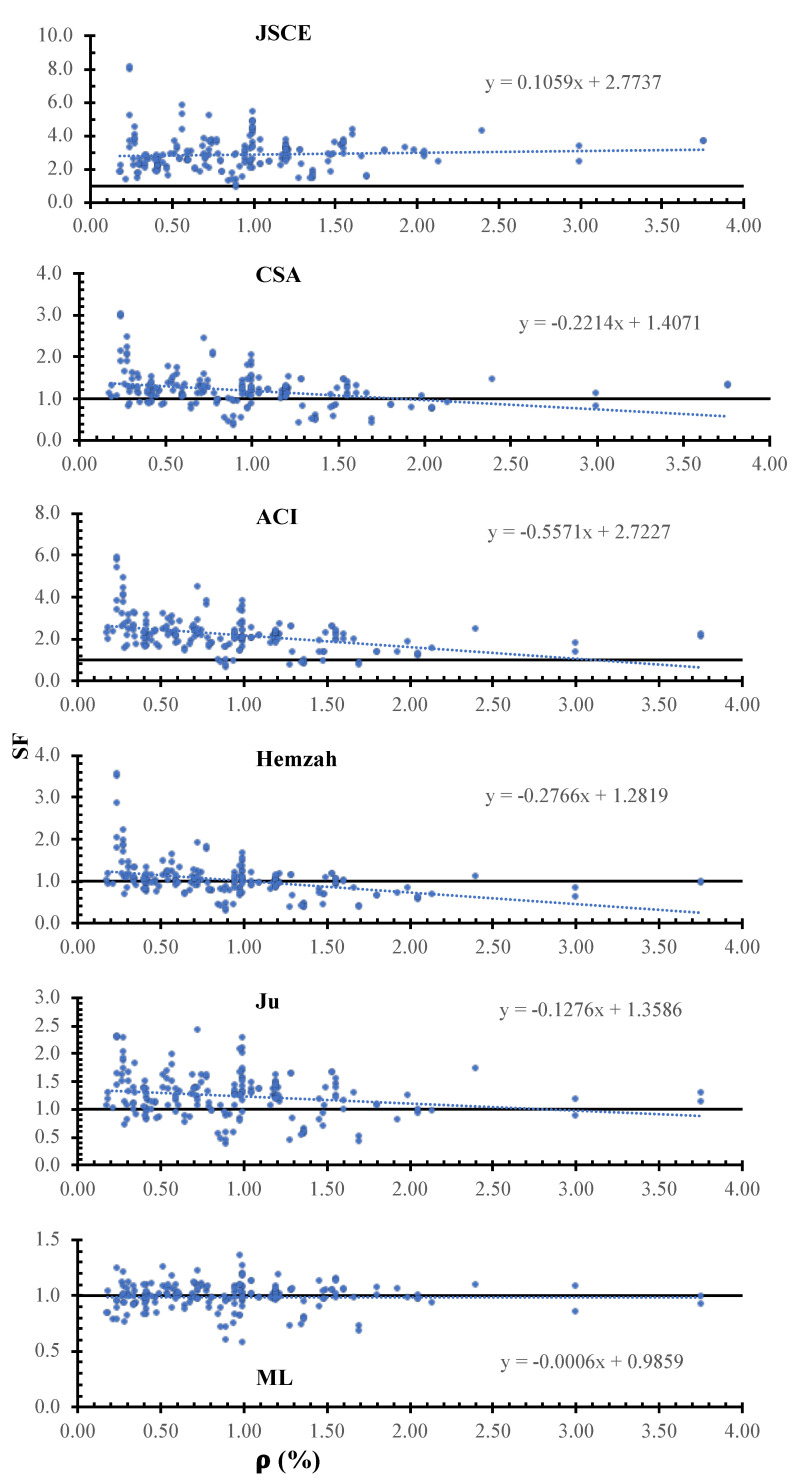
SF calculated using various models versus ρ.

Figure 10 shows the SF calculated using the JSCE, CSA, ACI, Hemzah, Ju, and ML models versus the flexure reinforcement ratio. In the figure, the inclinations of the trend line for the JSCE, CSA, ACI, Hemzah, and Ju models were $1059\times 10^{-4}$, $-2214\times 10^{-4}$, $-5571\times 10^{-4}$, $-2766\times 10^{-4}$, $-1276\times 10^{-4}$, and $-6\times 10^{-4}$. For all models, the safety decreases with the flexure reinforcement’s increase, except in the case of the JSCE and ACI models. The ML model has less scattering compared to the other models.

## 6. Conclusions

Several machine learning models were developed and evaluated using an extensive experimental database of 189 slab-column connections with FRP reinforcements. Although concluding remarks are limited to the range of parameter values in the database, a problem that can be solved with the testing of more slabs, the following can be concluded:

- A grid search with a 15-fold cross-validation was used to determine the optimal hyper-parameters of ML-based models during the training process.
- The comparison to the experimental data showed that the five ML-based models with the input variables and optimal hyper-parameters are fully capable of predicting the punching shear strength of FRP-RC slabs.
- The ensembled boosted model was found to be the most reliable and accurate model among all implemented machine learning models with the best accuracy: R^2^ = 0.97, RMSE = 71.963 kN, and MAE = 43.452 kN for the testing dataset. In addition, the boosted model predicted the actual strength more precisely and reliably compared to the existing design models. It minimized the variability of the traditional models with respect to the effective variables.
- For the most accurate model—the boosted ensemble—the effect of all input variables on the predicted Shear capacity was examined. Variables can be arranged from most to least influential as follows:
  1. The effective depth;
  2. The column dimensions;
  3. The flexure reinforcements;
  4. The longitudinal reinforcement modulus of elasticity;
  5. The concrete compressive strength.
- The proposed model has high accuracy and consistency and thus provides a reliable alternative to the existing strength models, which are inconsistent and have a high coefficient of variation. In addition, the interpretation results of the model reflect the importance and contribution of the parameters that influence the strength in the proposed model. Moreover, these findings confirm findings from concurrent research studies [70,71,72].

## Figures and Tables

**Table 3:** Experimental database for FRP-reinforced concrete slab-column connections.

Model	R^2^		RMSE (kN)		MAE (kN)		Training Time (secs)
Models	Train	Test	Train	Test	Train	Test	
**Linear **							
Normal	0.87	0.65	107.36	264.221	86.571	145.674	1.4376
Interaction	0.88	0.66	101.99	258.013	73.766	144.435	1.1043
Robust	0.9	0.63	95.409	240.48	76.542	116.275	0.97814
Stepwise	0.95	0.64	69.438	266.688	55.976	153.129	3.4838
**Tree**							
Fine	0.94	0.84	75.741	85.3446	52.98	60.8346	0.76536
Medium	0.93	0.44	78.387	357.974	57.683	216.808	0.63219
Coarse	0.82	0.63	128.12	214.858	112.3	117.108	0.50711
**Support Vector Machine**							
Linear	0.89	0.63	99.258	249.615	78.551	172.066	0.36803
Quadratic	0.88	0.71	104.89	214.858	62.374	109.986	1.7385
Cubic	0.77	0.49	143.89	341.117	97.009	219.475	1.6429
Fine Gaussian	0.79	0.59	137.11	250.681	102.54	169.551	1.5262
Medium Gaussian	0.96	0.69	57.815	236.587	46.092	109.372	1.4165
Coarse Gaussian	0.89	0.61	98.455	245.066	77.313	116.613	1.3137
**Ensembled Trees**							
**Boosted**	**0.98**	**0.97**	**44.12**	**71.963**	**35.95**	**43.452**	**1.1991**
Bagged	0.93	0.87	76.359	113.902	59.326	63.891	2.8842
**Gaussian Process Regression**							
Squared Exponential	0.95	0.93	68.981	150.097	53.354	77.068	0.95702
Marten 5/2	0.95	0.91	67.181	112.574	49.368	65.639	2.2757
Exponential	0.96	0.93	60.245	67.839	43.267	43.738	2.0637
Rational Quadratic	0.95	0.91	65.886	91.372	48.302	58.331	1.7053

**Table 4:** Statistical measures for the SF calculated using the existing models and the ML model.

Statistical Meaaure	JSCE	CSA	ACI	Hemzah	Ju	ML
**R${}^{2}$**	0.74	0.77	0.74	0.77	0.75	0.96
**RMSE**	375.94	169.58	305.58	157.87	181.30	64.23
**MAE**	274.36	112.52	222.62	100.98	121.25	37.97
**Mean**	2.87	1.20	2.20	1.02	1.24	0.99
**C.O.V**	36%	37%	39%	43%	32%	12%
**Lower 95%**	2.72	1.14	2.08	0.96	1.18	0.97
**Maximum**	0.85	0.34	0.62	0.28	0.36	0.57
**Minimum**	8.08	3.00	5.86	3.54	2.41	1.35

## Data Availability

All data are available within the manuscript.
